# TCMPG: an integrative database for traditional Chinese medicine plant genomes

**DOI:** 10.1093/hr/uhac060

**Published:** 2022-03-08

**Authors:** Fanbo Meng, Qiang Tang, Tianzhe Chu, Xianhai Li, Yue Lin, Xiaoming Song, Wei Chen

**Affiliations:** 1 State Key Laboratory of Southwestern Chinese Medicine Resources, Chengdu University of Traditional Chinese Medicine, Chengdu 611137, China; 2 Innovative Institute of Chinese Medicine and Pharmacy, Chengdu University of Traditional Chinese Medicine, Chengdu 611137, China; 3 School of Basic Medical Sciences, Chengdu University of Traditional Chinese Medicine, Chengdu 611137, China; 4 School of Pharmacy, Chengdu University of Traditional Chinese Medicine, Chengdu 611137, China; 5 School of Life Sciences, North China University of Science and Technology, Tangshan 063210, China

## Abstract

Because of their great therapeutic and economic value, medicinal plants have attracted increasing scientific attention. With the rapid development of high-throughput sequencing technology, the genomes of many medicinal plants have been sequenced. Storing and analyzing the increasing volume of genomic data has become an urgent task. To solve this challenge, we have proposed the **T**raditional **C**hinese **M**edicine **P**lant **G**enome database (TCMPG, http://cbcb.cdutcm.edu.cn/TCMPG/), an integrative database for storing the scattered genomes of medicinal plants. TCMPG currently includes 160 medicinal plants, 195 corresponding genomes, and 255 herbal medicines. Detailed information on plant species, genomes, and herbal medicines is also integrated into TCMPG. Popular genomic analysis tools are embedded in TCMPG to facilitate the systematic analysis of medicinal plants. These include BLAST for identifying orthologs from different plants, SSR Finder for identifying simple sequence repeats, JBrowse for browsing genomes, Synteny Viewer for displaying syntenic blocks between two genomes, and HmmSearch for identifying protein domains. TCMPG will be continuously updated by integrating new data and tools for comparative and functional genomic analysis.

## Introduction

Traditional Chinese medicine (TCM) has shown remarkable effects in the treatment of diseases [1–3] and has attracted increasing scientific attention worldwide. At present, more than 11 000 species of medicinal plants have been recorded in various TCM-related pharmacopoeias [4]. These medicinal plants constitute a huge library of natural organic compounds and have broad prospects for drug applications. With the development of modern science and technology, researchers have developed many commercially available drugs by purifying or modifying the natural components of plants [5]. As a result, medicinal plants have brought tremendous economic benefits to the pharmaceutical industry. Nonetheless, research on the modernization and standardization of medicinal plants still faces many challenges.

The continuous development of the high-throughput sequencing industry has promoted the study of medicinal plants and accelerated the shift from traditional research methods to the microscopic molecular level [6]. Through the efforts of genomic research, the genetic codes of many medicinal plants such as *Panax ginseng* [7, 8], *Artemisia annua* [9], and *Papaver somniferum* [10–12] have been successfully deciphered. The release of genome sequences has greatly promoted research on high-density DNA markers, gene annotation, and functional genomics. However, the proper storage, efficient processing, and integrative analysis of continuously increasing amounts of genomic data have become challenging tasks. Therefore, several databases have been developed to manage medicinal plant genomes, such as the Medicinal Plant Genomics Resource (MPGR, http://mpgr.uga.edu/), the Herbal Medicine Omics Database (HMOD) [13], and the Global Pharmacopoeia Genome Database (GPGD) [14]. However, most of these are expired or have limited information and functionality, hindering their wide application to medicinal plant genome analysis.

In the present study, we propose the **T**raditional **C**hinese **M**edicine **P**lant **G**enome database (TCMPG, http://cbcb.cdutcm.edu.cn/TCMPG/), which contains 195 genomes of 160 medicinal plants and basic information on 255 herbs. The database integrates five practical tools for genome analysis: BLAST, JBrowse, SSR Finder, Synteny Viewer, and HmmSearch. TCMPG provides not only the taxonomy and geographic distribution of plants but also related information on herbal medicines and relevant external links. We hope that TCMPG will become an important platform for comprehensive genomic data analysis of traditional Chinese medicinal plants.

## Database construction

### Acquisition of genomic data

In TCMPG, the genomic data for a medicinal plant consists of its reference genome and general feature format (GFF3), coding sequence (CDS), and protein sequence (PEP) files. We identified the available medicinal plant genome data through four steps. In the first step, we collected the Latin names and articles on all published, sequenced plants from plaBiPD (https://www.plabipd.de/index.ep). In the second step, we downloaded information on herbs from TCMID [15, 16] and HERB [17]. In the third step, the Latin names of the plants were used as a reference to obtain the intersection of the data acquired in the above two steps. In the fourth step, genomic data were identified by manual curation based on the links given in the article. If there were multiple versions for a medicinal plant, all versions were collected. Accordingly, 195 sets of high-quality genomic data were obtained for 160 medicinal plants.

### Supplements to plant and genome information

To enrich the data related to the plant species, we checked the taxonomy of each species at Wikipedia and downloaded representative images of most species. In addition, the chromosome number of each species was obtained from CCDB [18], and we acquired the distributions of most species in China from iPlant (www.iplant.cn).

We made a detailed record of each genome article, including the title, publication date, journal, and PMID. For each genome, we manually collected information from the articles, including the genome size, assembly level, and number of predicted genes. To show the landscape of the chromosomes in the genome, MCscan [19] (https://github.com/tanghaibao/jcvi/wiki/MCscan-(Python-version)) was used to display the gene density on each chromosome.

### Gene annotation and SSR search

We used InterProScan [20] to infer the functions of genes in the obtained genomes by searching against all databases contained in InterPro [21]. By collating the search results, we obtained the accession numbers and functional descriptions of the genes in the different databases. Then, we stored the annotation results of the genomes separately and linked the accession numbers to the corresponding databases.

Simple sequence repeats (SSRs) play important roles in herbal medicine variety identification, plant germplasm identification, genetic map construction, and genetic diversity analysis. To obtain SSRs in the medicinal plants, Batch_SSR_Finder.pl [22] written according to the Microsatellite identification tool (MISA) [23] was used with default parameters to identify SSR loci in all genes.

### Configuration of analysis tools

We integrated five useful bioinformatics tools into TCMPG: BLAST [24], JBrowse [25], SSR Finder, Synteny Viewer, and HmmSearch. To provide a user-friendly service, we built the BLAST service using the SequenceServer [26] application, a powerful BLAST front-end. The new version of JBrowse (JBrowse 2) was embedded into TCMPG to visualize all available genomes. To identify SSRs in sequences submitted by users, we developed the SSR Web interface modeled on the MISA page. The syntenic gene blocks between any two genomes were obtained using the jcvi.compara module of MCscan with default parameters. Protein domains were identified using the hmmsearch program in the HMMER (version 3.3.2) software package [27].

### Data integration and website construction

**Figure 1:**
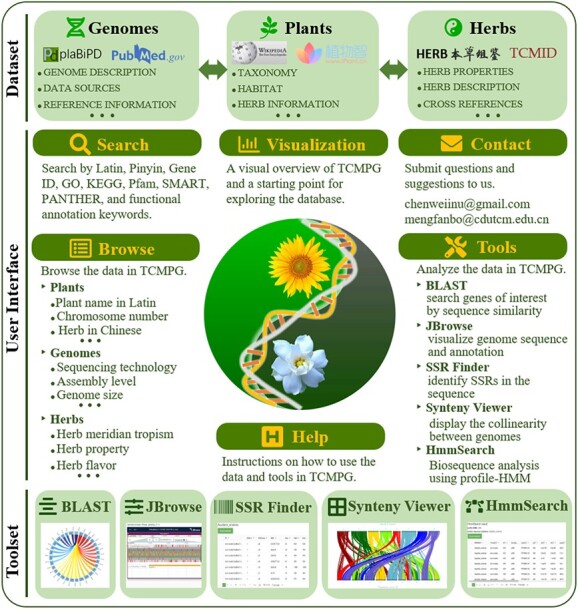
Workflow for the construction of the TCMPG database.

The TCMPG datasets are stored in a MySQL database. At present, 160 species, 195 genomes, and 255 herbs have been analyzed and organized in the MySQL database (Table S1). All data are interlinked by the Latin name of the species. An interactive Web interface was developed using the Django web framework and HTML; it allows users to easily access TCMPG and use analysis tools or obtain required information through any modern browser on their device. The workflow for the construction of TCMPG is shown in Fig. 1.

## Usage and access

### Main interface

**Figure 2:**
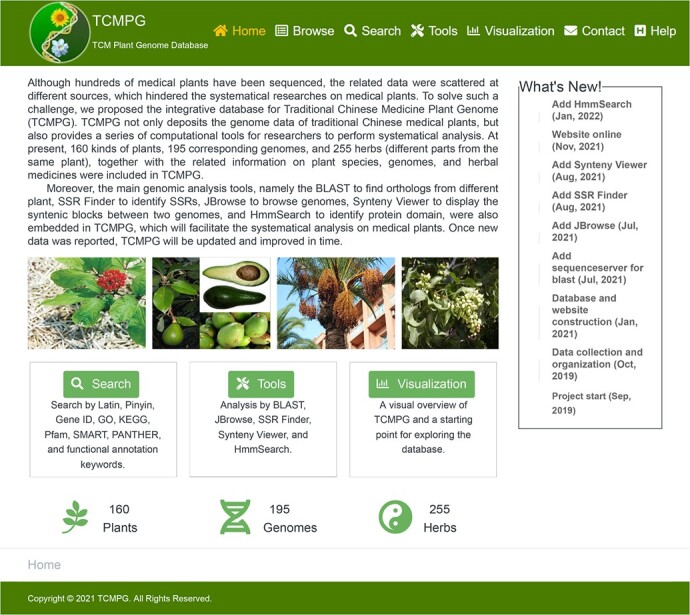
Homepage of TCMPG.

A well-organized and elegantly displayed TCMPG homepage was built at http://cbcb.cdutcm.edu.cn/TCMPG/. The current homepage mainly includes five parts: navigation bar, database introduction, sliding pictures of plants, common menu entrances, and other modules (Fig. 2). At the top of the home page, the navigation bar contains seven tabs: Home, Browse, Search, Tools, Visualization, Contact, and Help. Below the navigation bar is a brief introduction to the database, including an overview of the data and a brief introduction to the tools. The sliding pictures of plants are placed beneath the database introduction. The common menu entrances module located at the bottom of the homepage contains six entrances: Search, Tools, Visualization, Plants, Genomes, and Herbs.

**Figure 3:**
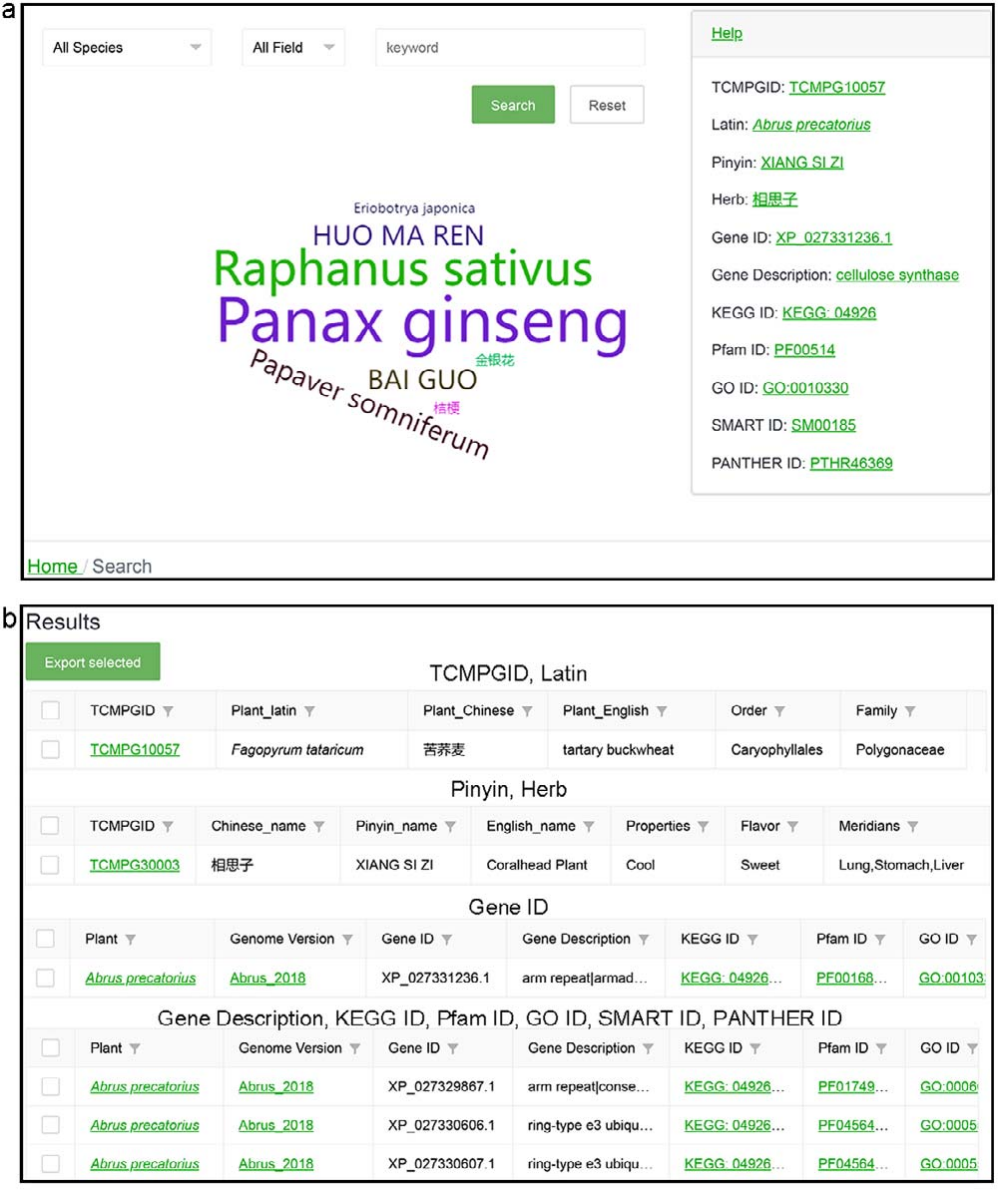
Search function in TCMPG. (a) Search interface. (b) Search result interface.

### Browse

In the browse module, we provide three types of data: plants, genomes, and herbs. Currently, there are 160 plants in TCMPG, associated with 195 different genome versions and 255 herbs (different parts from the same plant). Each type of data is presented in a table with basic information and external links providing detailed descriptions for each record. Users can browse the full list pages and the dedicated report card pages for the three types of entries assigned with unique TCMPG identifiers. On the full list page, users can filter the dataset with regard to several specific properties (e.g. species hierarchy, assembly level, and herbal properties) by using the interactive filters in the table header and browsing the subset of data that matches the filter properties. The report card pages provide more detailed information about the items, such as name and habitat (for plants), version and data source (for genomes), properties and description (for herbs), as well as internal links to other report card pages and external links to other databases (e.g. TCMID, HERB, and NCBI).

### Search

To help users quickly find data of interest, we deployed a separate search page in TCMPG (Fig. 3a). This page contains a search box consisting of two drop-down options and an input box. The first drop-down option contains the Latin names of all species in TCMPG and allows the user to directly select the species of interest and then click the search button to obtain the desired information. The second drop-down option is a search field where the user can select a field and then enter a keyword in the third input box to search. If no species is selected in the first drop-down option, the options that can be selected in the second drop-down option are TCMPGID, Latin, Pinyin, and Herb. When a species is selected in the first drop-down option, the options that can be selected in the second drop-down option are Gene ID, KEGG ID, GO ID, Pfam ID, SMART ID, etc. We recorded searches for plants and herbal medicines and displayed them in a word cloud image below the search box. Users can click the words in the word cloud to perform the corresponding search. Examples of search key words are also provided on the right side of the page.

The results page provides a brief description of the search in the form of a table (Fig. 3b). More details can be obtained by clicking the hyperlinks in the table.

**Figure 4:**
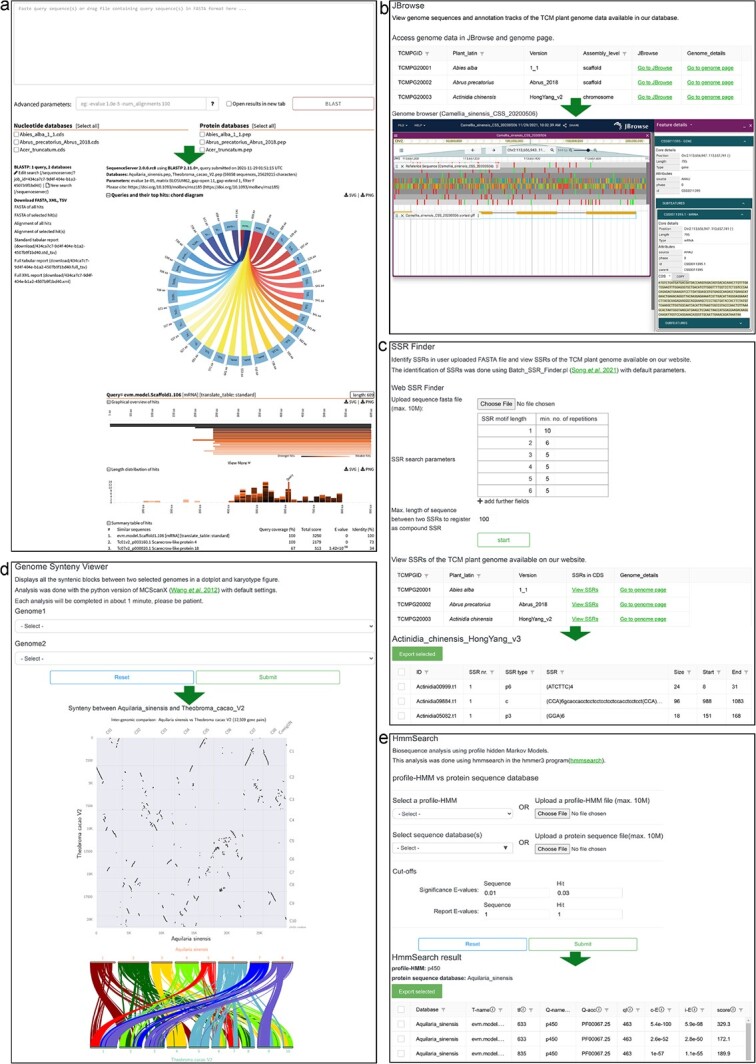
Analysis tools in TCMPG. (a) BLAST. (b) JBrowse. (c) SSR Finder. (d) Synteny Viewer. (e) HmmSearch.

*Tools*. To facilitate systematically analyzing the genomes of medicinal plants, several online analysis tools (BLAST, JBrowse, SSR Finder, Synteny Viewer, and HmmSearch) have been embedded in TCMPG (Fig. 4). They can be found under the Tools menu in the navigation bar or from the Tools page.

**Figure 5:**
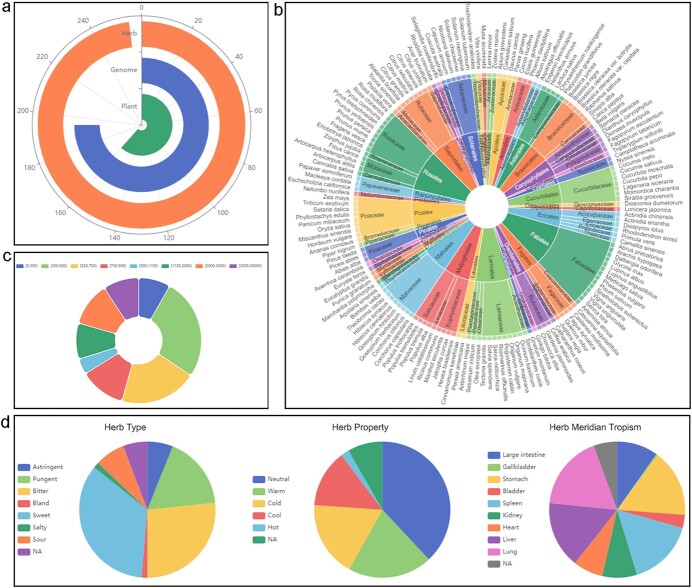
Data visualizations. (a) Polar coordinate bar chart showing statistics of all data. (b) Sunburst chart showing different hierarchies of plant taxonomy. The classification is arranged in concentric circles; from inside to outside are orders, families, and species. (c) Doughnut chart with rounded corners showing the proportions of genomes in different size ranges. (d) Pie chart showing the types, properties, and meridian tropisms of herbs.

*BLAST*. To provide a homology search function, we integrated SequenceServer (version 2.0.8) into TCMPG to enable BLAST analysis and visualize BLAST results (Fig. 4a). Users can paste the query sequence(s) or drag a file containing query sequence(s) in FASTA format into the input box, then select the database for homology search. These databases are all established based on the CDS and PEP sequences of genomes stored in TCMPG. The BLAST programs (BLASTN, BLASTP, BLASTX, tBLASTN, and tBLASTX) are automatically set up according to the type of query sequence and the selected database. In addition to using the default parameters, users can also modify parameters in the option box, such as e value, score, and output format, according to their research aims. The alignment results between query sequences and object sequences are presented using a variety of visualization methods and are available for download in vectorial (SVG) and PNG formats.

*JBrowse*. In the JBrowse module, we use a table to list all the genomes that can be browsed (Fig. 4b). All genome sequences and GFF3 annotations have been imported into JBrowse. Users can query the genome sequence of each chromosome or scaffold by scrolling and zooming. They can also click on a gene and view its detailed information, such as gene ID, sequence, intron-exon structure, location, and length, in the righthand column. In addition, using the high flexibility and customization of JBrowse, users can upload their data for visualization and compare them with datasets in TCMPG.

*SSR Finder*. The SSR Finder page consists of two sub-modules (Fig. 4c). The top sub-module is the Web SSR Finder, which allows users to upload their sequences and set parameters to identify SSRs. The bottom sub-module is a table containing the SSRs identified from the coding sequences of the genome deposited in TCMPG. The results include the number, type, sequence, size, and start/end position of SSRs in the query sequence.

*Synteny Viewer*. This tool is able to explore homology comparisons, evolution, and whole-genome duplication (WGD) events. After selecting the query and target genomes and clicking the submit button, the collinearity landscape between the selected genomes will be obtained (Fig. 4d). The collinear relationships between genomes are shown in two formats. The first one is a dot-plot depicting the collinear genes between the two genomes. The other is the karyotype between the two genomes, where the collinear genes are connected by Bezier curves.

*HmmSearch*. To facilitate the analysis of gene families, we have configured HmmSearch in TCMPG. Users can select the profile Hidden Markov Models (profile-HMMs) already stored in TCMPG or upload a profile-HMM of interest to identify domains in the genomes deposited in TCMPG or in a self-uploaded protein sequence. In the cut-off box, users can change the reporting and inclusion thresholds to control the number of hits. After the “Submit” button is clicked, the results reporting the domain hits table will be shown on a new page (Fig. 4e).

### Visualization

To display the data included in TCMPG, we created several interactive data visualizations (Fig. 5) using JavaScript and ECharts (version 5.2.0, https://echarts.apache.org, 2021). Users can view the data through the visualization buttons on the navigation bar. This will serve as a starting point from which users can explore the database.

At the top of the visualization page, we present statistics on the three types of data in TCMPG (plants, genomes, and herbs) using a polar bar chart (Fig. 5a). Users can click on a bar to jump to the sub-category graph of the corresponding data. For the plant data, we used a Sunburst chart to show different taxonomic levels. Users can click on any section to expand it and can use the button below the chart to retrieve the corresponding entries (Fig. 5b). For the genome data, we use a doughnut chart with rounded corners to show the proportions of genomes in different size ranges (Fig. 5c). We display the herb data in three separate pie charts based on their flavors (sour, salty, sweet, bitter, pungent), properties (cold, hot, warm, cool, even), and meridian tropisms (lung meridian, liver meridian, etc.) (Fig. 5d). Users can click on any part of the genome or herb charts to retrieve the corresponding entries.

### Contact and help

We have created a form in the contact module for users to give feedback on issues and suggestions. In addition, our contact email address is also given on the contact page to help users get in touch with us quickly and easily. To ensure ease of use, we have provided instructions on how to use the main modules on the help page.

### Discussion and future perspectives

With the rapid development of sequencing technology, a large number of species with medicinal value have been sequenced. Accordingly, several databases have been developed to deposit the genomes of one or more medicinal plants, such as the Ginseng Genome Database [31], MPGR (http://mpgr.uga.edu/), and HMOD [13]. However, most of these databases were constructed several years ago and have not been updated or have even become inaccessible. Moreover, genomic data in these databases cannot easily be utilized by researchers. With this in mind, we collected and processed the available medicinal plant genomes and established the Traditional Chinese Medicine Plant Genomes database (TCMPG).

In TCMPG, we collected 195 sets of genomes for 160 plants, involving 255 Chinese herbal medicines. It should be pointed out that some sequenced medicinal plants, such as *Panax notoginseng* [32], *Eucommia ulmoides* [33], and *Scutellaria baicalensis* [34], were not included in TCMPG due to their unreleased genome annotations or restrictions on dataset usage.

More importantly, we have implemented five popular and powerful bioinformatics tools in TCMPG, which not only facilitate the analysis and visualization of the genomes in TCMPG but also allow the users to analyze their own in-house data.

As more and more genomes are released, we will continue to collect new medicinal plant genome data and store them in TCMPG. In addition to herbs, animals and fungi are also invaluable to medicine. However, their sequencing data are still scattered in different databases, such as NCBI [28], NGDC [29], and EnsemblFungi [30]. Hence, further efforts are also needed to develop databases for medicinal animals and fungi.

## Conclusions

By integrating 195 medicinal plant genomes and corresponding herbal information, we constructed the Traditional Chinese Medicine Plant Genome (TCMPG) database, which is a comprehensive and freely accessible resource for medicinal plants. Popular and powerful bioinformatics tools have been implemented in TCMPG, allowing users to conveniently analyze and visualize the genomes from multiple perspectives. We hope that TCMPG will become a user-friendly website for researchers on medicinal plants.

## Acknowledgements

This work was supported by the National Natural Science Foundation of China (Nos. 31771471 and 32172583).

## Author contributions

WC and XS conceived and led the research. FM collected the data and constructed the database. QT, TC, XL, YL, and XS helped with the database design. QT and TC contributed to data collection. YL and XS joined constructive discussions. FM, XS, and WC wrote the paper. All authors read and approved the final manuscript.

## Data availability

All the data associated with this study are provided at http://cbcb.cdutcm.edu.cn/TCMPG/ and in Supplementary Table S1.

## Conflict of interest

The authors declare that they have no conflicts of interest.

## Supplementary data

Supplementary data is available at *Horticulture Research * online.

## Supplementary Material

Web_Material_uhac060
